# Supplementary material for: Hibernation Patterns of the European Hedgehog, Erinaceus europaeus, at a Cornish Rescue Centre
Source: Animals (Basel). 2020 Aug 14;10(8):1418. doi: 10.3390/ani10081418 (PMC7459883; doi:10.3390/ani10081418)
Supplement: Supplementary file 1 [file animals-10-01418-s001.zip › Animals-867385_Suppl_2.pdf]

Prickles & Paws Hedgehog Rescue
www.pricklesandpaws.org / hello@pricklesandpaws.org
01637831299 / 07926576164

Prickles & Paws
Hedgehog Rescue

Supplementary material 2.

Hibernation protocol 2015-16 winter

Suitability for hibernation:

Is the hedgehog:

- Completely finished all courses of medication? (plus at least 7 days clear to ensure fully metabolised)
- Clinically healthy, with no skin conditions, parasites (faecal sample clear), injuries or ailments
- Over 600g in weight?

  AND
- In good body condition (spherical in shape when curled up). If oval or rear end is triangular when curled then the hedgehog is still underweight for its size and should not be allowed to hibernate. NB. Weight must always be considered alongside condition; a 600g hedgehog could still be very thin or underweight for its size.

Pre-hibernation monitoring:

For each hedgehog awake:

- Observe inside of hutch daily for activity levels and indications of hibernation.
- Hedgehogs in outdoor hutches that are suitable for hibernation, but can not yet be released (e.g. because of unsuitable weather or lack of suitable location) are to be weighed once or twice weekly whilst awake. Record details on individual's health record.
- When showing signs of reduced activity, declining food consumption and dark green faeces, weighing and other disturbance should be halted. Record details on health record. If activity resumes before hibernation, resume weekly weighing.

In-hibernation monitoring:

For each hibernating hedgehog:

- Record date of start of hibernation on health record.
- Minimise disturbance to the hutch.
- Observe inside of hutch daily, but do not open door without good reason.
- Record start and end of any periods of activity (visible movement, disturbance to nesting material or food, presence of faeces).
- During periods of arousal in mid-winter, replace food and water if necessary.
- After 6 weeks of hibernation weigh the hedgehog. Gently remove the hedgehog, place on digital scales, weigh to nearest gram, record on health record. Refresh nesting material and food if necessary.
  - If hedgehog is < 400g, arousal is to be induced by handling and stroking. If the hedgehog does not show any signs of activity, the following night it can be moved to an inside hutch and if necessary gradually warmed with heat pads.
  - If hedgehog is >400g replace in hutch to continue hibernation. Add note to health record if disturbance during weighing induced arousal.
- Final arousal – If hedgehog arouses naturally towards end of winter, treat initally as for spontaneous arousal and do not weigh immediately. If they remain awake for more than 5 days and/or are increasingly active and feeding regularly, then treat as final arousal; weigh and record details on health record.
- When conditions are suitable and a suitable location is available, do final weigh and health check prior to release.

Post-hibernation monitoring:

- If arousal is induced artificially, weigh the hedgehog daily to ensure that they are eating and gaining weight. It can sometimes take a few days for them to start eating properly.
- If final arousal happens naturally before spring, monitor weight on a weekly or twice-weekly basis to ensure that weight is maintained and that the hedgehog does not become obese. Release the individual as soon as the weather conditions allow and the release can be organized with the finder or one of our release sites.
