# Supplementary material for: Hibernation Patterns of the European Hedgehog, Erinaceus europaeus, at a Cornish Rescue Centre
Source: Animals (Basel). 2020 Aug 14;10(8):1418. doi: 10.3390/ani10081418 (PMC7459883; doi:10.3390/ani10081418)
Supplement: Supplementary file 1 [file animals-10-01418-s001.zip › Animals_867385_Suppl_1.pdf]

Supplementary material 1.

Husbandry records and changes in body-weight for hedgehogs studied at Prickles and Paws rescue centre during the winters of 2015-16 and 2016-17. The sets of hibernatory weights are intentionally

Year	ID	Sex	Life stage on admission	Admitted	Admittance weight (g)	Reason for admission	Parasitic burden	Treatments administered	Hibernated	# Days captive before hibernation	Pre-hibernation weight (g)	Mid-hibernation weight (g)	End-hibernation weight (g)	Weight-loss (g)	Final arousal	Total hibernation period (nights)	# Spontaneous arousals	Mean duration of spontaneous arousals (nights)	Release weigh[illegible]
2015-16	227/15	Male	Juvenile	05/10/2015	177	Out in day, very underweight	None	None	17/12/2015	73	691	590 (30/01/16) 571 (27/02/16)	524	167	05/04/2016	110	2	1	759
2015-16	286/15	Female	Juvenile	27/10/2015	318	Trapped in chicken coop for unknown time	*Crenosoma striatum*, + 3 ticks	Levacide, Baytril	07/01/2016	72	776	-	887	-111	25/01/2016	18	3	2.67	921
2015-16	272/15	Female	Juvenile	18/10/2015	281	RTA, old injuries partially healed	*Crenosoma striatum*, Fluke	Levacide, Profender	05/01/2016	79	774	-	774	0	01/02/2016	27	6	2.5	775
2015-16	292/15	Female	Juvenile	01/11/2015	290	Trapped in a pit	*Capillaria* spp., 3 ticks	Ivomec, Baytril	27/12/2015	56	697	622 (30/01/16) 597 (22/02/16)	582	115	01/04/2016	96	15	1.67	580
2015-16	320/15	Female	Juvenile	15/11/2015	336	Out in day	*Crenosoma striatum*, *Capillaria* spp., +9	Levacide, Ivomec	23/12/2015	38	591	-	480	111	30/01/2016	38	6	1.67	623
2015-16	256/15	Male	Juvenile	14/10/2015	262	Dog attack	*Capillaria* spp.	Ivomec, Baytril	12/12/2015	59	657	542 (30/01/16)	495	162	01/04/2016	111	5	1	685
2015-16	280/15	Female	Hoglet	22/10/2015	207	Out in day, shallow wounds	None	Synulox	03/01/2016	73	552	-	487	65	01/02/2016	29	1	3	594
2015-16	212/15	Female	Juvenile	27/09/2015	276	Out in day, flystrike, wounds	*Crenosoma striatum*	›vacide, Baytril, Loxico	21/12/2015	85	867	810 (10/01/16)* weighed early as aroused and active in hutch	760	107	06/02/2016	47	9	1.44	878
2015-16	235/15	Female	Hoglet	07/10/2015	107	Out in day, very hypothermic	None	Baytril	07/01/2016	92	585	-	518	67	01/02/2016	25	1	1	616
2015-16	288/15	Male	Juvenile	29/10/2015	257	Out in day	*Crenosoma striatum*, + 2 ticks	Levacide	23/12/2015	55	633	-	521	112	31/01/2016	39	4	2.25	808
2015-16	255/15	Male	Hoglet	13/10/2015	157	Out in day	*Crenosoma striatum*	Levacide	10/01/2016	89	725	-	738	-13	23/01/2016	13	2	2	920
2015-16	336/20	Male	Juvenile	27/11/2015	271	Out in day	*Crenosoma striatum*	Baytril, Levacide	07/01/2016	41	717	-	737	-20	06/02/2016	30	3	1.67	980
2015-16	308/20	Male	Juvenile	09/11/2015	273	Out in day, very underweight	*Crenosoma striatum*	Levacide	20/12/2015	41	729	674 (30/01/16)	615	114	02/04/2016	104	10	1.1	708
2015-16	223/15	Female	Hoglet	03/10/2015	104	Out in day, no sign of mum	*Crenosoma striatum*	Levacide	17/12/2015	75	631	-	493	138	30/01/2016	44	1	1	672
2015-16	224/15	Male	Hoglet	04/10/2015	114	Out in day	*Crenosoma striatum*, *Capillaria* spp.	Levacide, Ivomec, Baytril	05/01/2016	93	884	805 (10/02/16)	734	150	05/04/2016	91	4	1	704
2015-16	152/15	Male	Hoglet	27/08/2015	77	Out in day, no sign of mum	None	None	10/11/2016	441	555	-	488	67	29/11/2016	19	1	1	697
2015-16	296/15	Male	Juvenile	02/11/2015	331	Out in day	*Capillaria* spp. +1 tick	Ivomec	06/01/2016	65	884	945 (02/02/16)	970	-86	22/02/2016	47	5	3.8	981
2015-16	271/15	Male	Juvenile	18/10/2015	185	Out in day, underweight	*Crenosoma striatum*, Fluke	Levacide, Profender	06/01/2016	80	677	-	625	52	26/01/2016	20	6	1.17	781
2015-16	317/20	Male	Juvenile	13/11/2015	226	RSPCA, no history provided	*Crenosoma striatum*, +8 ticks	Levacide	06/01/2016	54	713	618 (6/02/16)	604	109	05/04/2016	90	11	1.09	755
2015-16	175/15	Male	Juvenile	08/09/2015	195	Out in day, underweight, severe ringworm	None	aytril, Loxicom, Imaver	20/12/2016	469	844	752 (14/02/16)	770	74	23/02/2017	65	4	1.5	741
2015-16	264/15	Female	Hoglet	17/10/2015	141	Out in day	*Crenosoma striatum*, *Capillaria* spp.	Baytril, Ivomec, Levacide	06/01/2016	81	661	-	611	50	24/01/2016	18	3	1.67	829

Year	ID	Sex	Life stage on admission	Admitted	Admittance weight (g)	Reason for admission	Parasitic burden	Treatments administered	Hibernated	# Days captive before hibernation	Pre-hibernation weight (g)	Mid-hibernation weight (g)	End-hibernation weight (g)	Weight-loss (g)	Final arousal	Total hibernation period (nights)	# Spontaneous arousals	Mean duration of spontaneous arousals (nights)	Release weight [illegible]
2016-17	363/16	Female	Juvenile	02/11/2016	214	Out in day	*None*	None	13/01/2017	72	N/A	N/A	N/A	N/A	17/03/2017	63	3	1.666667	768
2016-17	350/16	Male	Juvenile	29/10/2016	201	Out in day	*Crenosoma striatum*, *Capillaria* spp.	Levacide, Ivomec, Baytril	27/01/2017	90	N/A	N/A	N/A	N/A	09/03/2017	41	6	4	968
2016-17	374/16	Male	Juvenile	06/11/2016	366	Severe ringworm	*Capillaria* spp.	Ivomec, Baytril, Imaverol	09/01/2017	64	N/A	N/A	N/A	N/A	15/03/2017	65	6	2.333333	1028
2016-17	426/16	Male	Juvenile	30/11/2016	247	Out in day	*Crenosoma striatum*, *Capillaria* spp.	Levacide, Ivomec, Baytril	16/03/2017	106	N/A	N/A	N/A	N/A	05/04/2017	20	4	1	759
2016-17	370/16	Female	Juvenile	04/11/2016	285	Very underweight	*Crenosoma striatum*, *Capillaria* spp.	Levacide, Ivomec	15/01/2017	72	N/A	N/A	N/A	N/A	15/02/2017	31	3	4.666667	854
2016-17	390/16	Male	Juvenile	12/11/2016	273	From a vet. Hypothermic, no other history provided	*Crenosoma striatum*, *Capillaria* spp.	Levacide, Baytril	17/02/2017	97	N/A	N/A	N/A	N/A	03/04/2017	45	5	2.2	788
2016-17	427/16	Female	Juvenile	30/11/2016	348	Out in day	*Crenosoma striatum*, *Capillaria* spp., + 1 tick	Levacide, Ivomec	16/01/2017	47	N/A	N/A	N/A	N/A	20/03/2017	63	2	1.5	719
2016-17	324/16	Male	Juvenile	21/10/2016	224	Out in day	*Crenosoma striatum*, *Capillaria* spp., + 2 ticks	evacide, Ivomec, Baytr	29/01/2017	100	N/A	N/A	N/A	N/A	03/04/2017	64	4	2.75	793
2016-17	448/16	Female	Juvenile	15/12/2016	315	Out in day	*Capillaria* spp.	Ivomec	28/01/2017	44	N/A	N/A	N/A	N/A	21/03/2017	52	4	1.25	741
2016-17	397/16	Female	Juvenile	15/11/2016	306	Small for time of year	None	None	16/01/2017	62	N/A	N/A	N/A	N/A	21/01/2017	5	1	0	713
2016-17	438/16	Female	Juvenile	06/12/2016	370	Out in day	*Capillaria* spp.	Ivomec	08/01/2017	33	N/A	N/A	N/A	N/A	18/03/2017	69	2	2	972
2016-17	333/16	Male	Hoglet	23/10/2016	120	Admitted with very unwell mum	None	None	22/01/2017	91	N/A	N/A	N/A	N/A	02/04/2017	70	2	1	660
2016-17	299/16	Female	Hoglet	08/10/2016	197	RSPCA no history provided	*Crenosoma striatum*, *Capillaria* spp.	Levacide, Ivomec	19/02/2017	134	N/A	N/A	N/A	N/A	19/03/2017	28	4	2	922
